# Assessment of the excitation–inhibition ratio in the Fmr1 KO2 mouse using neuronal oscillation dynamics

**DOI:** 10.1093/cercor/bhae201

**Published:** 2024-05-20

**Authors:** Renate Kat, Klaus Linkenkaer-Hansen, Marthe A Koopmans, Simon J Houtman, Hilgo Bruining, Martien J H Kas

**Affiliations:** Groningen Institute for Evolutionary Life Sciences, University of Groningen, Nijenborgh 7, 9747 AG, Groningen, The Netherlands; Department of Integrative Neurophysiology, Center for Neurogenomics and Cognitive Research, Vrije Universiteit Amsterdam, De Boelelaan 1085, 1081 HV, Amsterdam, The Netherlands; Groningen Institute for Evolutionary Life Sciences, University of Groningen, Nijenborgh 7, 9747 AG, Groningen, The Netherlands; Department of Integrative Neurophysiology, Center for Neurogenomics and Cognitive Research, Vrije Universiteit Amsterdam, De Boelelaan 1085, 1081 HV, Amsterdam, The Netherlands; Department of Child and Adolescent Psychiatry, Amsterdam UMC, University of Amsterdam, Meibergdreef 9, 1105 AZ, Amsterdam, The Netherlands; Groningen Institute for Evolutionary Life Sciences, University of Groningen, Nijenborgh 7, 9747 AG, Groningen, The Netherlands

**Keywords:** electroencephalogram, biomarker, autism spectrum disorder, psychiatry, resting state

## Abstract

In vitro and ex vivo studies have shown consistent indications of hyperexcitability in the Fragile X Messenger Ribonucleoprotein 1 (Fmr1) knockout mouse model of autism spectrum disorder. We recently introduced a method to quantify network–level functional excitation–inhibition ratio from the neuronal oscillations. Here, we used this measure to study whether the implicated synaptic excitation–inhibition disturbances translate to disturbances in network physiology in the Fragile X Messenger Ribonucleoprotein 1 (Fmr1) gene knockout model. Vigilance-state scoring was used to extract segments of inactive wakefulness as an equivalent behavioral condition to the human resting-state and, subsequently, we performed high-frequency resolution analysis of the functional excitation–inhibition biomarker, long-range temporal correlations, and spectral power. We corroborated earlier studies showing increased high-frequency power in Fragile X Messenger Ribonucleoprotein 1 (Fmr1) knockout mice. Long-range temporal correlations were higher in the gamma frequency ranges. Contrary to expectations, functional excitation–inhibition was lower in the knockout mice in high frequency ranges, suggesting more inhibition-dominated networks. Exposure to the Gamma-aminobutyric acid (GABA)-agonist clonazepam decreased the functional excitation–inhibition in both genotypes, confirming that increasing inhibitory tone results in a reduction of functional excitation–inhibition. In addition, clonazepam decreased electroencephalogram power and increased long-range temporal correlations in both genotypes. These findings show applicability of these new resting–state electroencephalogram biomarkers to animal for translational studies and allow investigation of the effects of lower-level disturbances in excitation–inhibition balance.

## Introduction

Neuronal excitation–inhibition (E/I) imbalances have been put forward as a framework to understand pathophysiological elements of neurodevelopmental disorders, such as autism spectrum disorder (ASD) (Rubenstein and Merzenich 2003; Nelson and Valakh 2015; Dickinson et al. 2016; Lee et al. 2017; Sohal and Rubenstein 2019; Zhao et al. 2022). Indeed, animal models of ASD and other disorders have shown alterations of E/I ratio regulation at cellular and synaptic levels that may be contributing to E/I imbalances in neuronal networks (Antoine et al. 2019; Iascone et al. 2020); however, noninvasive measures for assessments of how E/I disturbances affect mass–brain activity are currently lacking (Sohal and Rubenstein 2019). Recently, a quantitative measure was developed, which provides an estimate of network-level E/I ratio by applying a computational algorithm to spectro-temporal characteristics of human resting-state electroencephalogram (EEG) oscillations (Bruining et al. 2020). This functional E/I (fEI) outcome is expected to be close to 1 when calculated on a signal generated by a network operating in an E/I-balanced state. The authors showed a reduction in the fEI after using the Gamma-aminobutyric acid (GABA) potentiator Zolpidem and demonstrated responsiveness of the biomarker to bumetanide in patients with ASD (Bruining et al. 2020; Juarez et al. 2021).

The fEI measure was developed on human EEG data, and it is not yet known whether the algorithms can also be applied to rodent EEG data. In the present study, we assessed the fEI and 2 other measures of neuronal oscillation dynamics, namely power and long-range temporal correlations (LRTC), in a genetic mouse model for Fragile X syndrome and ASD (Fragile X Messenger Ribonucleoprotein 1 (Fmr1) knockout [KO]), for which neuronal hyperexcitability was previously suggested. Decreased neuronal inhibition as well as increased neuronal excitation have been shown in these KO animals based on ex vivo electrophysiological recordings and in vitro assessments of GABA signaling (D’Hulst et al. 2006; Gibson et al. 2008; Adusei et al. 2010; Olmos-Serrano et al. 2010; Deng et al. 2011; Paluszkiewicz et al. 2011; Rotschafer and Razak 2014; Sabanov et al. 2017). In addition to assessing baseline differences between the genotypes, we also studied how the neuronal oscillation dynamics responded to the administration of pharmacological compounds acting on the GABA and glutamate systems. This pharmacological intervention aimed to confirm that in mice increasing the inhibitory tone is associated with reductions in the fEI. Furthermore, we aimed to assess potential differences in responses to the pharmacological compounds between the genotypes, since paradoxical increases in arousal in response to benzodiazepines (GABA agonists; Marrosu et al. 1987; Bruining et al. 2015) and opposite directionality of the effects of riluzole (combined GABA agonist and glutamate antagonist) have been reported in patients compared to controls (Ajram et al. 2017).

## Materials and methods

Two separate experiments were performed in this study. In the first experiment wild-type (WT) and KO mice underwent EEG recordings and assessment of the network properties without further intervention. To get a better understanding of the network features, this experiment was followed by an experiment in which we performed a pharmacological intervention on the E/I balance. Both experiments had a similar methodology and are therefore described together. It is pointed out when there were differences between the experiments.

### Mice

Original breeding females from the FMR1 KO2 line (Mientjes et al. 2006) were generously provided by the Netherlands Institute for Neuroscience (Amsterdam, NL) and crossed with C57/BL6J from Envigo. Heterozygous females were crossed with WT and KO males to obtain FMR1^−/y^ (KO, experiment 1 and 2: *n* = 12) and FMR1^+/y^ (WT, experiment 1: *n* = 10, experiment 2: *n* = 12) male littermates. Since this was the first time we tried to implement the fEI algorithm in a mouse experiment, samples sizes were based on previous experience with event related potential (ERP) studies in mice (Perenboom et al. 2020; Kat et al. 2021; Kat and Kas 2022). Deoxyribonucleic acid for genotyping was extracted from ear clips using a lysis buffer (100 mM Tris pH 8.0, 200 nM NaCl, 5 nM ethylenediaminetetraacetic acid) with proteinase K (0.2 mg/ml). Reverse transcription polymerase chain reaction was performed using the following primers: reverse transgene (5′ GCC TCA CAT CCT AGC CCT CTA C 3′), reverse internal control (5′ CCC ACA AAG TTGA TTC CCC AGA 3′), forward transgene/internal control (5′ CCC ACT GGGA GAG GAT TAT TTG GG 3′). Mice were weaned at 3 to 4 wk of age. Until surgery at 12 (experiment 1) or 8 (experiment 2) wk, animals were housed with 4 animals from different nests and mixed genotypes (2 KO and 2 WT) in a cage. Mice were kept on a 12:12 light–dark cycle with ad libitum access to food and water. All experiments were performed in accordance with the European Directive 2010/63/EU and according to a research protocol approved by the local animal welfare body. All efforts were made to minimize discomfort of the experimental animals. Findings are reported in adherence to the Animal Research: Reporting of In Vivo Experiments (ARRIVE) guidelines (Percie du Sert et al. 2020).

### Electroencephalography

**Fig. 1:**
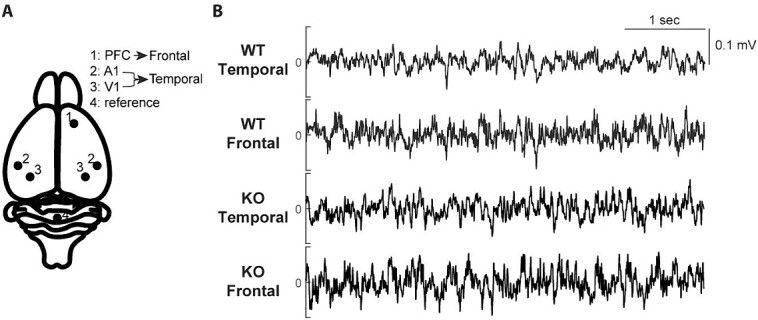
Increased power in Fmr1 KO animals in the beta- and gamma-frequency ranges. A) Schematic representation of the electrode locations. Electrodes above the auditory (A1) and visual (V1) cortices were averaged for a temporal cortex signal. B) Example EEG-traces of a WT and a KO mouse. The signal shows 5 s of oscillations after 1 Hz high-pass and 100 Hz low-pass filtering.

EEG recordings were performed in the wireless multichannel TaiNi EEG system (Tainitec, London, UK). Animals underwent stereotactic EEG implantation surgery under isoflurane anesthesia (0.8% to 1.5%, in oxygen-enriched air) and local lidocaine anesthesia at 12 (experiment 1) or 8 (experiment 2) wk of age. Custom-designed implants (ND & Associates, Berkshire, UK) were used, which slightly differed in design between the first and second experiment. In the first experiment, 6 epidural screw electrodes (0.7 mm, Antrin miniature specialties, Fallbrook, United States) were implanted. One electrode was placed above the prefrontal cortex (bregma +2.6 mm anterior, +1.6 mm lateral) and bilateral electrodes were placed above the primary visual cortex (bregma −3.5 mm posterior, ±3.0 mm lateral) and the primary auditory cortex (bregma −3 mm posterior, ±3.2 mm lateral; Fig. 1A). A reference screw electrode was placed centrally on the cerebellum. Two electromyography (EMG) electrodes were placed under the neck muscles. In the second experiment, silver ball tip (approximately 0.2 mm diameter) electrodes were implanted above the left primary auditory cortex instead of the screw electrodes. Coordinates were based on the Allen Brain Atlas (Allen Institute for Brain Science 2011). Both the electrodes and the implant were fixated on the skull using dental cement (RleyX unicem aplicap cement, 3M, Minneapolis, United States). Painkilling was applied by a subcutaneous injection of Carprofen (5 mg/kg) both before the start of the surgery as well as 24 h post-surgery. After waking up from anesthesia 1 KO animal from experiment 2 had an epileptic seizure followed by cardiac arrest.

After surgery, animals were pair-housed with mixed genotypes in semi-separating cages. In the middle of the cage a see-through plexiglass separator, with a fissure at head-height, was placed. This separator allowed for visual, auditory, olfactory, and some extent of somatosensory contact between pair-housed animals, but prevented cage mates from damaging each other’s implant. Mice had access to a paper roll, nesting material, and a plexiglass house. In the second week of operative recovery animals were habituated to handling, short head fixation via holding the implant with plyers (necessary for connecting the transmitter), and connecting and wearing the transmitter. EEG recordings started after a 14-d recovery period. EEG recordings were performed between 15 and 27 (experiment 1) or 10 and 13 (experiment 2) wk of age. Before the first resting-state recording animals were tested in auditory and visual ERP paradigms which have been published elsewhere (Kat and Kas 2022).

EEG signals were recorded with an approximately 19,525 Hz (experiment 1) or 1,085 Hz (experiment 2) sampling rate and 9,700 Hz low-pass and 0.35 Hz high-pass online filters in the TaiNiLive software (Tainitec, London, United Kingdom). Activity of the animals during EEG recordings was recorded via passive infrared (PIR) motion sensors with the Micro1401 (Cambridge Electronic Devices, Cambridge, United Kingdom). Digital pulses were sent to the EEG system every minute for later synchronization of the EEG data with the movement data.

### Pharmacological intervention (experiment 2)

In order to challenge the E/I balance, a pharmacological intervention was performed with both a GABA-A agonist (Clonazepam, CLZ, Duchefa Farma, lot: 006180.06) and a glutamate antagonist (MPEP-hydrochloride, Sigma-Aldrich, M5435, lot: BGBC0044V). The pharmacological compounds and their dosages were selected based on pharmacokinetic properties. Too short half-lives would not ensure significant activity during the 4-h long EEG recordings, while too long half-lives are not suitable in a cross-over design. CLZ has a half-life of 1.5 to 2 h in young animals (Barnhill et al. 1990) and has shown effects on EEG spiking rates in mice up to 4 h after administration (Handforth et al. 2005). The high CLZ dosage was chosen in such a way that it does not yet exert sedative effects (Brooks and Peever 2011; Han et al. 2012) to avoid large differences in the amount of resting-state data. The low CLZ dosage was considered sufficient to expect effects in the EEG, as this dosage has previously shown to exert strong effects on spiking rates in mouse EEG (Zhu et al. 2018). MPEP was chosen as glutamate antagonist, as it is metabolized faster than other antagonists like CTEP (Lindemann et al. 2011), but at a dosage of 30 mg/kg does keep bio-availability for several hours (Lindemann et al. 2011; Westmark et al. 2018). Animals received a high (0.1 mg/kg) and low (0.025 mg/kg) dose CLZ, MPEP (25 mg/kg) and vehicle in a cross-over design over the course of 4 wk, with a 1-wk wash-out period between treatments. Dosages were on purpose chosen to be relatively low, as we expected that too high dosages would prevent us from detecting possible differences between the genotypes in sensitivity or reactivity to the compounds. The order of treatments was randomized over the cages using Blindr (2018, U8N WDX). The KO and WT animal in 1 cage received the same treatment at the same time, assuring a counterbalanced design over the genotypes. Saline (0.9%) was used as a vehicle for both compounds. Since CLZ is known to have lower solubility, it was dissolved overnight. All compounds were made a week before the onset of experiments. Saline and CLZ were stored in the refrigerator (4°C), while MPEP was stored in the freezer (−20°C). Because of the different storing places, experimenters could not be blinded for the compound during the experiments. Experimenters were blinded for the animal’s genotype. Compounds were injected intraperitoneally (10 mL/kg), after which the TaiNi transmitter was connected. Scoring of the resting state was performed from the moment the experimenters left the experimental room where 2 cages (4 animals) were recorded in parallel. The delay between the injection of the first animal in the room and the moment of leaving the room was 6 to 12 min. Animals were not habituated to intraperitoneal injections.

### Resting-state recordings

Resting-state recordings were performed in the home cage. Four hours of undisturbed baseline recording were collected in the second half of the light phase (5 h—1 h before lights off), in dim light (~50 lux). Mice had access to food and a water gel (HydroGel [70-01-5022], Bio Services, Uden, The Netherlands) during recordings. To extract phases of “resting-state”, vigilance state scoring was performed in the Spike2 software (v10, Cambridge Electronic Devices). Five-second epochs were scored using a 70 Hz low-pass filtered signal of the prefrontal cortex electrode. An epoch was labeled as resting state, if no slow-wave sleep was detected in the frontal signal (defined as the absence of high-amplitude waves with a frequency below 4 Hz), nor any motor activity (assessed by a combination of a PIR movement sensor as well as the EMG). Resting state could never directly follow an epoch which was scored as sleep, to reduce the chance of scoring rapid eye movement sleep as resting state. Resting-state recordings were scored in randomized order (using the RAND function in R), with the assessor being blinded for the genotype, the compound, and the consecutive week. One animal (KO) from experiment 1 was excluded because the quality of the EMG was too low to reliably score the resting state.

### Analysis

Data analysis was performed in Matlab (v2018a and v2018b, MathWorks, Natick, MA, United States). For experiment 1, recordings were down-sampled to 1,000 Hz after which the resting-state epochs were extracted. Gaps in the signals, as a result of the wireless transmitter not being able to connect to the antennas, were detected. Epochs containing gaps of more than 10 ms were excluded from the analysis (signal-loss artifact), while gaps of less than 10 ms were interpolated by drawing a straight line from the last datapoint before to the first datapoint after the gap. Subsequently, manual artifact rejection was performed to exclude epochs containing high-amplitude deflections, high-frequency noise, or abundance of artificial spikes. Artifact rejection was performed blinded for genotype and compound. None of the animals exceeded the a priori set threshold of rejection of 50% of the original data. However, 1 WT animal from experiment 1 was excluded from the analysis because it did not reach the a priori set minimum of 80 s of resting-state EEG (Supplementary Fig. S1 and S2). In the second experiment for this reason the MPEP recording of a WT and 1 MPEP and 1 clonazepam low-dose recording of 2 different KO mice were excluded. The amount of available resting-state data was compared between genotypes and pharmacological interventions with a Mann–Whitney *U*-test and a Kruskal–Wallis test, respectively.

To obtain power, fast Fourier transform (FFT) was applied to the included data epochs and power was extracted as the squared absolute value of the data length-normalized FFT product. Power was averaged in 1-Hz bins between 1 and 99 Hz. Detrended fluctuation analysis (DFA) and fEI were computed as previously described (Bruining et al. 2020). Continuous frequency spectra were analyzed between 1 and 99 Hz in 1-Hz bins, by applying a hamming window bandpass filter for every frequency bin (Houtman et al. 2021). The amplitude envelope was obtained by performing a Hilbert transform on the demeaned filtered signal. For the DFA, the maximum window length was set to 20 s, the minimum window length was 4 s for the delta and theta band (<8 Hz), 2 s for the alpha band (8 to 13 Hz), and 1 s for the beta and gamma bands (>13 Hz). The window overlap was set to 0.8. For all windows the cumulative sum of the demeaned signal was taken and detrended before the fluctuation (i.e. standard deviation) was calculated. A regression was fitted through the mean fluctuation of every window length over all window lengths. The DFA exponent was defined as the exponent of this regression (Linkenkaer-Hansen et al. 2001; Hardstone et al. 2012). For the fEI analysis a window length of 5 s with 0.8 overlap was used. For every window, the mean amplitude of cumulative sum of the demeaned signal was calculated. The signal in every window was amplitude normalized by dividing it by the mean amplitude and subsequently detrended. The fluctuation (i.e. standard deviation) of these detrended normalized signals was correlated with the mean amplitude. The fEI component is defined as 1 minus the correlation coefficient of this correlation. Windows which were an outlier, as tested with the generalized extreme studentized deviate test for outliers, in either mean amplitude or fluctuation were not included in the correlation. Functional E/I was only computed when the DFA exponent of a signal was above 0.6 because the fEI method assumes a signal close to the E/I-balanced regime where oscillations have significant LRTC (Bruining et al. 2020).

### General additive mixed modeling

Generalized Additive Mixed Modeling (GAMM) was used for the statistical analysis of the data. GAMM is relatively new in the field of EEG research, but has proved to be suitable on simulated data and has been used before in several studies (Kryuchkova et al. 2012; De Cat et al. 2015; Meulman et al. 2015;Smith and Kutas 2015; Tremblay and Newman 2015). An insightful tutorial paper on these type of analyses has also been published using articulatory time-series data (Wieling 2018). GAMMs can also be used to model courses of our network measures over the full frequency spectrum. GAMM allows for optimal use of the frequency resolution of the EEG data, as data does not need to be diminished to a single mean amplitude or area under the curve value for specific power bands. GAMM is an extended version of linear mixed effects modeling, which can also model nonlinear patterns like the ones found in EEG data. GAMM fits a number of nonlinear basis functions over the data while finding a balance between minimizing the error, but also preventing overfitting. Extensive explanations on how GAMM works and is applied to EEG data have been published before and will thus not be described here (Smith and Kutas 2015; Tremblay and Newman 2015).

The power, DFA, and fEI spectra were modeled over frequency per genotype (WT and KO) and pharmacological compound (saline, clonazepam low, clonazepam high, and MPEP; experiment 2) using the *bam()* function from the *mcgv* package (v1.8-40) (Wood 2011) in R (v4.2.1, R core team, 2018) with single animals as experimental unit. The models were given 40 basis-functions to model the data; post-hoc checks showed this to be sufficient. Random effects over frequency were added to the model for individual animals (separately per pharmacological compound for experiment 2). When working with series data (i.e. frequency series in this case), it is important to assess the autocorrelation in the residuals of the model. When the series correlates with itself at different lags (meaning that the outcome at frequency x correlates to the outcome at frequency x + 1, x + 2, etc.), the assumption of independence of each measurement point will lead to an underestimation of standard errors of the effects. Significant autocorrelation was present in the frequency spectra of the DFA and the fEI, which was corrected for using a correlation parameter estimated as the correlation at lag 1 in a model fitted without correlation correction. This correlation parameter was then used to apply an autoregressive model on the residuals (using the *rho* argument of the *bam* function). No strong autocorrelation was present in the power spectra and correction therefore not necessary. For the data of the second experiment model comparison based on the Akaike information criterion (AIC) was used to assess whether adding the pharmacological compounds and subsequently the interaction between the pharmacological compounds and the genotype as a factor to the model significantly improves the fit of the model [using the *compareML()* function of the *itsadug* package (v2.4.1); van Rij et al. 2015]. Separate models were built for the 3 outcome measures (power, DFA, and fEI) as well as the recording location. An overview of the final models can be found in the Supplementary Materials.

Difference waves from the fitted frequency spectra were computed using the *itsadug* package to compare the genotypes with each other and the various pharmacological compounds against saline. Frequency ranges where the confidence intervals (CIs) did not cross zero were considered significant. For the comparison of the genotypes 95% CIs were used, corresponding to an α-threshold of 0.05. For the comparison of the pharmacological compounds 98.3% CIs were used, to correct for the triple comparison (98.3% CI corresponds to an α-threshold of 0.0167 as would be required with Bonferroni correction). Separate models were constructed for the data from the frontal and temporal cortex for all network measures.

The descriptive statistics of the number of resting-state epochs is presented as median (interquartile range). Figures on the main outcomes present mean ± 95% CI shading. An exception are the difference plots for the pharmacological compounds, where 98.3% CI are shown.

## Results

Neuronal oscillation dynamics were assessed in 2 independent experiments. An initial experiment only compared WT and KO mice (experiment 1), while a follow-up experiment included pharmacological interventions in both genotypes (experiment 2). We developed EEG-based definitions of inactive wakefulness, to study the best possible equivalent vigilance states of human resting-state EEG. After artifact rejection, we obtained an equal number of 5-s epochs classified as resting state to perform the analyses in WT (experiment 1: 55 [46.5–61] epochs, corresponding to 275 s; experiment 2: 43 [32–57.5] epochs, corresponding to 215 s) and KO animals (experiment 1: 40 [28–47.5] epochs, corresponding to 200 s, *P* = 0.068, Supplementary Fig. S1; experiment 2: 40 [22–57.5] epochs, corresponding to 200 s, *P* = 0.238, Supplementary Fig. S2). The number of resting-state epochs did differ between the pharmacological interventions, with a significant increase in the number of resting-state epochs after CLZ high administration compared to saline and MPEP (saline: 32 [21.5–47]; CLZ Low: 40 [31–48]; CLZ High: 57 [43–77.5]; MPEP: 39 [26–49.3], *P* = 0.007, Supplementary Fig. S2a).

### Fmr1 KO mice show reproducible alterations in neuronal oscillation dynamics

**Fig. 2:**
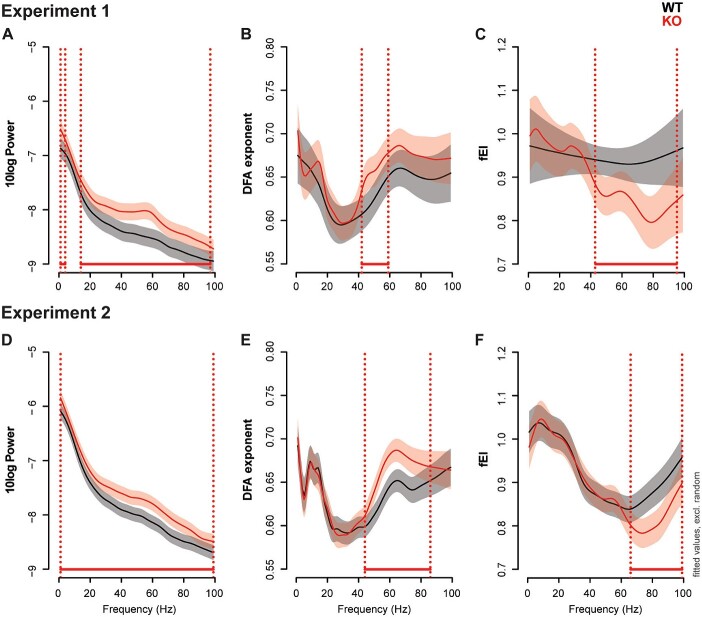
Fmr1 KO2 mice show reproducible alterations in temporal cortex network function. Resting-state EEG was extracted from baseline recordings to assess network functionality in the Fmr1 KO2 model. EEG power (A and B), LRTC (C and D), and the fEI ratio (E and F) were computed in 1-Hz frequency bins in the 1 to 99 Hz range. GAMMs were used to model the effect of genotype (and pharmacological exposure in experiment 2). The figures show the fitted values of the effect of genotype excluding random effect factors of the electrodes on the temporal cortex. For experiment 2 (D to F), the fitted values from the saline condition are shown for both WT and KO mice. Frequency ranges, where the confidence bands of the difference curve between WT and KO (not shown) do not cross zero, were considered significant and are marked with horizontal lines above the frequency-axes in the figures. Experiment 1: N_WT_ = 9, N_KO_ = 11; experiment 2: N_WT_ = 12, N_KO_ = 11.

To assess neuronal oscillation dynamics of the Fmr1 KO2 mice, power, LRTCs, and fEI were computed from resting-state data obtained in 2 independent experiments. Overall, results were more pronounced in temporal compared to frontal cortex recordings. In the temporal cortex signals, significant genotype effects were found in all 3 outcome measures in both experiments. EEG power was higher in KO animals over the whole frequency spectrum. In experiment 1, this difference was only significant in the delta, beta, and gamma frequency ranges (1 to 4 Hz, 14 to 97 Hz), but in experiment 2 the higher power in KO animals was found to be significant over the full frequency spectrum (1 to 99 Hz; Fig. 2A). The LRTCs, measured as the DFA exponent, were also higher in the gamma frequency ranges in KO animals (experiment 1: 42 to 59 Hz; experiment 2: 44 to 86 HZ; Fig. 2B). The fEI was lower in KO animals, again in the ranges of the gamma frequency (experiment 1: 43 to 95 Hz; experiment 2: 66 to 99 Hz; Fig. 2C), indicating inhibition-dominated network activity. Although the high degree of overlap between the frequency ranges which show genotype differences in the 2 experiments indicates robustness of the findings, due to the exploratory nature of these experiments the results should be interpreted carefully for the specific frequency ranges were genotype effects were present in only 1 of the 2 experiments (power: 5 to 13 Hz, DFA: 60 to 86 Hz, fEI: 43 to 65 Hz).

In the frontal oscillations, genotype effects were mainly present in EEG power, which was higher in KO animals in the beta and gamma frequency ranges (experiment 1: 28 to 93 Hz; experiment 2: 19 to 94 Hz; Supplementary Fig. S3a). The LRTCs were unaltered in the frontal signals of KO animals (Supplementary Fig. S3b). A specific reduction in fEI was seen in KO animals between 61 to 64 Hz in experiment 2, but not in experiment 1 (Supplementary Fig. S3c).

### Clonazepam modifies neuronal oscillation dynamics in the same direction as KO of the Fmr1 gene

**Fig. 3:**
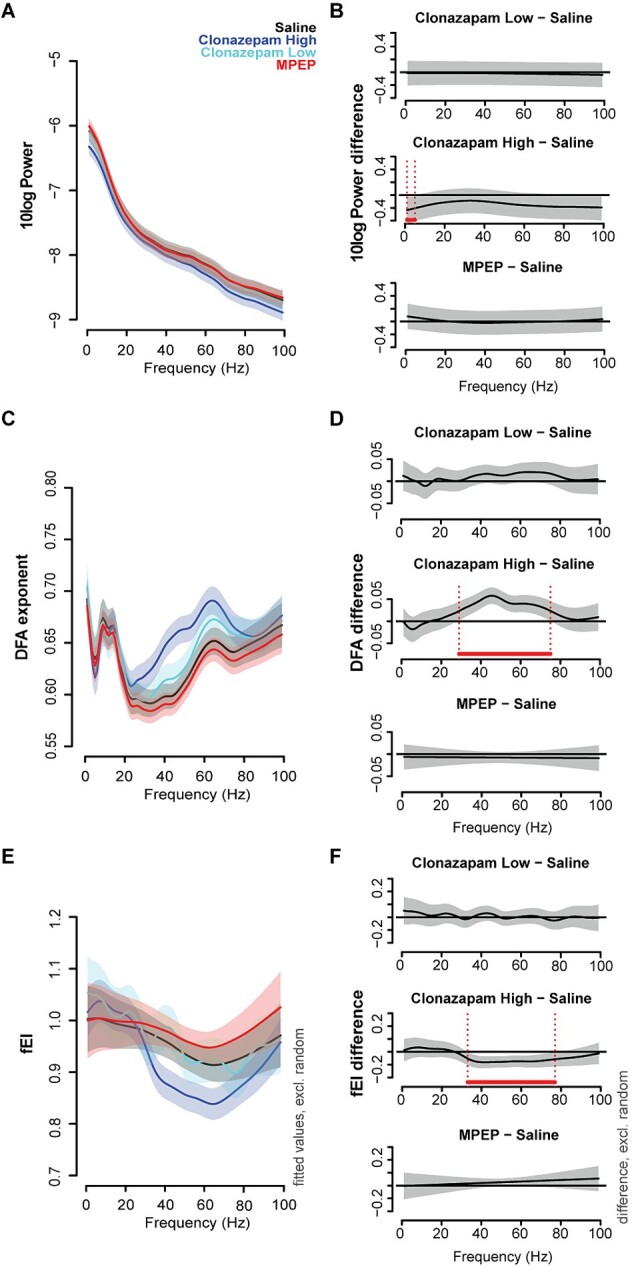
High-dose clonazepam, but not MPEP, affects the oscillatory brain dynamics in the temporal cortex independent of genotype. Resting-state EEG was extracted from baseline recordings to assess network functionality in the Fmr1 KO2 model after administration of low (0.1 mg/kg) or high (0.25 mg/kg) dose clonazepam and MPEP (25 mg/kg). EEG power (A and B), LRTC (C and D), and the fEI ratio (E and F) were computed in 1-Hz frequency bins in the 1 to 99 Hz range. GAMMs were used to model the effect of genotype and pharmacological exposure. The figures show the fitted values, without random effects of the temporal cortex recording electrodes. Since there was no interaction between genotype and pharmacological exposure, the figure shows the fitted values for WT mice. The fitted values for KO mice are shown in Supplementary Fig. S4. Frequency ranges, where the confidence bands of the difference wave between saline and the pharmacological compounds do not cross zero, were considered significant and marked with horizontal lines above the frequency-axes in the figures. (B, D, and F). *n* = 12 (N_MPEP_ = 11).

To confirm that in mice, like in human EEG data, lower fEI is also associated with inhibition-dominated dynamics, in a second experiment we exposed the animals to pharmacological compounds increasing the relative inhibitory tone, either by increasing GABA-A activity (CLZ) or by inhibiting glutamate activity (MPEP). We administered a low and a high dose of CLZ and a single dose of MPEP in a cross-over design. The neuronal oscillations were significantly affected by the administration of the high dose of CLZ only. Compared to saline, administration of CLZ decreased power in the delta ranges (1 to 5 Hz, Fig. 3B), and respectively increased and decreased the DFA exponent and the fEI in the gamma frequency ranges of the temporal cortex signals (DFA: 29 to 75 Hz, fEI: 33 to 77 Hz, Fig. 3D and F).

In order to assess whether there was an interaction effect between the genotype and the pharmacological compounds, we tested whether the models better described the data when adding this interaction to the model. The addition of an interaction term between genotype and pharmacological compound exposure did not increase the percentage of variance in the data that the models explained (Table 1). Besides, the AIC value of the models, which is a measure for how well a model fits the data by weighing the complexity (number of parameters) and the explained variance, indicated that the models were not improved by adding the interaction. Smaller AIC values indicate an improvement of the fit of a model, but higher AICs were found for all models with the interaction (Table 1). These results indicate that both genotypes showed the same response to the pharmacological interventions. The effect of the pharmacological intervention on the power, DFA, and fEI spectra in KO animals is shown in Supplementary Fig. S4.

**Table 1:** Addition of an interaction term between genotype and pharmacological exposure does not improve the models fit to the data. The explained variance of the models fitted on the oscillations recorded from the temporal cortex showed minimal increases after the addition of the interaction term. The AIC value, a weighted factor between explained variance and model complexity, increased after addition of the interaction while decreases in AIC indicate model improvement.

	Explained variance of model without interaction	Explained variance of model with interaction	ΔAIC (AIC model with interaction – AIC model without interaction)
Power	92.6%	92.7%	+36.1
DFA	87.0%	87.4%	+4.4
fEI	72.6%	72.2%	+19.7

The effects were again less pronounced in the signals from the frontal electrodes. For power and fEI, adding the interaction between genotype and pharmacological exposure did not improve the fit of the models (power: ΔAIC +26.7, fEI: ΔAIC +19.7), indicating that the oscillations in the frontal cortex showed the same response to the pharmacological exposure in both genotypes. Alike the data recorded from the temporal cortex, high dose CLZ decreased power in the gamma ranges (67 to 99 Hz); however, it did not affect the fEI (Supplementary Fig. S5 b and d). For the DFA, not only the addition of the interaction, but also the addition of pharmacological exposure as an independent predictor did not improve the model (ΔAIC +2.6). The effects of pharmacological exposure were therefore not further analyzed in the frontal DFA data.

## Discussion

In this exploratory study, we assessed neuronal oscillation dynamics of the Fmr1 KO2 mouse. We first optimized the detection of the resting-state segments in mouse EEG using a combination of EEG, EMG, and a behavioral sensor. Compared to WT, KO mice were found to have more inhibition-dominated network activity in the high-frequency ranges, as shown by lower fEI ratios. Exposure to the GABA-agonist CLZ confirmed that increasing the inhibitory tone in the neuronal network was associated with higher LRTCs and a lower fEI ratios both in WT and KO mice.

In an effort to increase the translational validity of our results, we performed our analyses specifically on resting-state segments. Most of the EEG studies in the Fmr1 KO2 animals assessed power in a freely behaving state irrespective of the vigilance state of the animal, although some studies did control for the presence of movement (Lovelace et al. 2018; Lovelace et al. 2020; Pirbhoy et al. 2020; Schaefer et al. 2021) and 1 study actually dissociated awake and sleep-like immobility using video analysis (Hooper et al. 2021). The selection of resting-state segments by manner of manual vigilance state scoring is labor-intensive; however, being able to distinguish wakeful inactivity from sleep is essential because of the large differences in oscillatory dynamics between the various vigilance states. While using this optimized dissociation between sleep and resting-state strategy, we replicated the previously reported increase in gamma power in the KO mice (Lovelace et al. 2018; Wen et al. 2019; Jonak et al. 2020; Kozono et al. 2020; Lovelace et al. 2020; Pirbhoy et al. 2020; Hooper et al. 2021; Schaefer et al. 2021), indicating that these previous findings were likely not confounded by differences in activity levels or vigilance states between genotypes. The selection of resting-state segments specifically may however be more critical for a translationally valid assessment of LRTCs and fEI.

The finding of reduced fEI, i.e. more inhibition-dominated activity, in the Fmr1 model may seem contradictory to what is known about E/I ratio alterations in this genetic rodent model from ex vivo and in vitro observations. Interestingly, electrophysiological recordings in brain slices of the Fmr1 model showed evidence for a hyperexcitable phenotype (Gibson et al. 2008; Olmos-Serrano et al. 2010; Deng et al. 2011; Sabanov et al. 2017; Antoine et al. 2019; Zhao et al. 2022). Additionally, GABA availability and expression of GABA receptor subunits have been found to be decreased in these KO mice (D’Hulst et al. 2006; Adusei et al. 2010; Olmos-Serrano et al. 2010; Paluszkiewicz et al. 2011; Sabanov et al. 2017; Zhao et al. 2022). Furthermore, we confirmed the increased gamma power which has been previously shown in large-scale network activity in vivo using EEG (Lovelace et al. 2018; Wen et al. 2019; Jonak et al. 2020; Kozono et al. 2020; Lovelace et al. 2020; Pirbhoy et al. 2020; Hooper et al. 2021; Schaefer et al. 2021). Such broadband increases in gamma power have been associated with higher levels of excitatory activity (Yizhar et al. 2011; Cho et al. 2015; Vogt et al. 2015; Sohal and Rubenstein 2019). In our study, gamma power indeed seemed to be decreased after pharmacologically increasing inhibitory the tone; however, this reduction was not significant. On the other hand, the effects of the pharmacological intervention with a GABA-agonist were in line with the idea that an increase in inhibitory drive is associated with a lower fEI ratio in mouse EEG data, similar as was found in human studies (Bruining et al. 2020). It is, therefore, unlikely that the contradiction between the cellular and large-scale brain network E/I findings are explained by an invalidity of the fEI measure in mice. Rather, the inconsistency in findings between cellular, power, and fEI measurements (an indication of increased excitation in cellular findings and gamma power vs an indication of increased inhibition in large-scale network findings), emphasizes the complexity of the excitation inhibition balance and our limited understanding thereof. Seemingly counterintuitive reductions in fEI ratio (between 12 to 24 Hz) were also recently reported in patients with STXBP1 encephalopathy (Houtman et al. 2021) and tuberous sclerosis complex (Juarez-Martinez et al. 2022). The discrepancies between the in vivo, ex vivo, and in vitro findings in the Fmr1 and other models illustrate the challenge of comparing different levels of E/I balance interrogation such as activity in local “disconnected” networks in slice preparations versus the quantification of mass brain activity in EEG. Additionally, they highlight the complexity of the E/I balance, which is thought to be tightly regulated and deviations can be compensated at many biological levels (Turrigiano 2011; Barral and D’Reyes 2016; Sohal and Rubenstein 2019). To start elucidating the relations between synaptic, circuit, and large-scale brain network E/I ratios, future studies should exploit the extensive methodological possibilities in mice compared to human studies. Correlation of measures of synaptic excitation and inhibition with the fEI measure after genetic or pharmacological interventions could elucidate the underlying mechanisms which drive changes in fEI measure. Additionally, applying techniques like opto- and pharmacogenetics, with which synaptic and circuit-level E/I ratios can be experimentally targeted while measuring large-scale brain network E/I, could provide further validation as well as understanding of how EEG power, DFA, and fEI respectively represent large scale excitation and inhibition in the brain. Until these types of experiments have been performed, the results of this study should be interpreted carefully. Nevertheless, do the results of this study challenge our current understanding of E/I balance alterations in neurodevelopmental disorders, and highlight that we do not yet grasp the full complexity of the E/I balance, both in health and disease.

Taken together, this study showed that the Fmr1 KO2 mice showed repeatable alterations in neuronal oscillation dynamics, which suggest inhibition-dominated activity in high-frequency oscillations. The results indicate that changes in the E/I of large-scale brain networks cannot simply be inferred one-on-one from synaptic findings. Future studies will have to confirm the exploratory results and elucidate how the findings of synaptic hyperexcitability can be linked to inhibition-dominated fEI in the Fmr1 KO2 mice. Further parallel characterization of the EEG measures used in this study in rodents and human disorder equivalents can determine their value as translational biomarkers.

## Supplementary Material

20240419_Supplementary_materials_bhae201

## Data Availability

The data that support the findings of this study are openly available in the OSF repository at [https://osf.io/as7cz/].
