# Supplementary material for: Assessment of the excitation–inhibition ratio in the Fmr1 KO2 mouse using neuronal oscillation dynamics
Source: Cereb Cortex. 2024 May 20;34(5):bhae201. doi: 10.1093/cercor/bhae201 (PMC11107376; doi:10.1093/cercor/bhae201)
Supplement: 20240419_Supplementary_materials_bhae201 [file 20240419_supplementary_materials_bhae201.docx]

**Supplementary materials**

GAMM models:

Primary GAMM models contained a smooth over frequency by genotype (GT). Model comparison based on AIC was used to assess whether adding a smooth over frequency by pharmacological compounds (Comp) and an interaction between genotype and pharmacological compound improved the fit of the model (the lower AIC, the better the fit of the model). Autocorrelation correction (ACC) was added to the models when necessary.

**Experiment 1**

Temporal power ~ s(Freq, by = GT, k = 40) + GT + s(Freq, AnID, bs = "fs", m=1)

Frontal power ~ s(Freq, by = GT, k = 40) + GT + s(Freq, AnID, bs = "fs", m=1)

Temporal DFA ~ s(Freq, by = GT, k = 40) + GT + s(Freq, AnID, bs = "fs", m=1) *+ ACC*

Frontal DFA ~ s(Freq, by = GT, k = 40) + GT + s(Freq, AnID, bs = "fs", m=1) *+ ACC*

Temporal fEI ~ s(Freq, by = GT, k = 40) + GT + s(Freq, AnID, bs = "fs", m=1) *+ ACC*

Frontal fEI ~ s(Freq, by = GT, k = 40) + GT + s(Freq, AnID, bs = "fs", m=1) *+ ACC*

**Experiment 2**

Temporal Power ~ s(Freq, by = Comp, k = 40) + Comp + s(Freq, by = GT, k = 40) + GT + s(Freq, AnID, by = Comp, bs = "fs", m=1) *+ ACC*

Frontal Power ~ s(Freq, by = Comp, k = 40) + Comp + s(Freq, by = GT, k = 40) + GT + s(Freq, AnID, by = Comp, bs = "fs", m=1) *+ ACC*

Temporal DFA ~ s(Freq, by = Comp, k = 40) + Comp + s(Freq, by = GT, k = 40) + GT + s(Freq, AnID, by = Comp, bs = "fs", m=1) *+ ACC*

Frontal DFA ~ s(Freq, by = GT, k = 60) + GT + s(Freq, AnID, by = Comp, bs = "fs", m=1) *+ ACC*

Temporal fEI ~ s(Freq, by = Drug, k = 40) + Drug + s(Freq, by = GT, k = 40) + GT + s(Freq, AnID, by = Drug, bs = "fs", m=1) *+ ACC*

Frontal fEI ~ s(Freq, by = Drug, k = 40) + Drug + s(Freq, by = GT, k = 40) + GT + s(Freq, AnID, by = Drug, bs = "fs", m=1) *+ ACC*

**
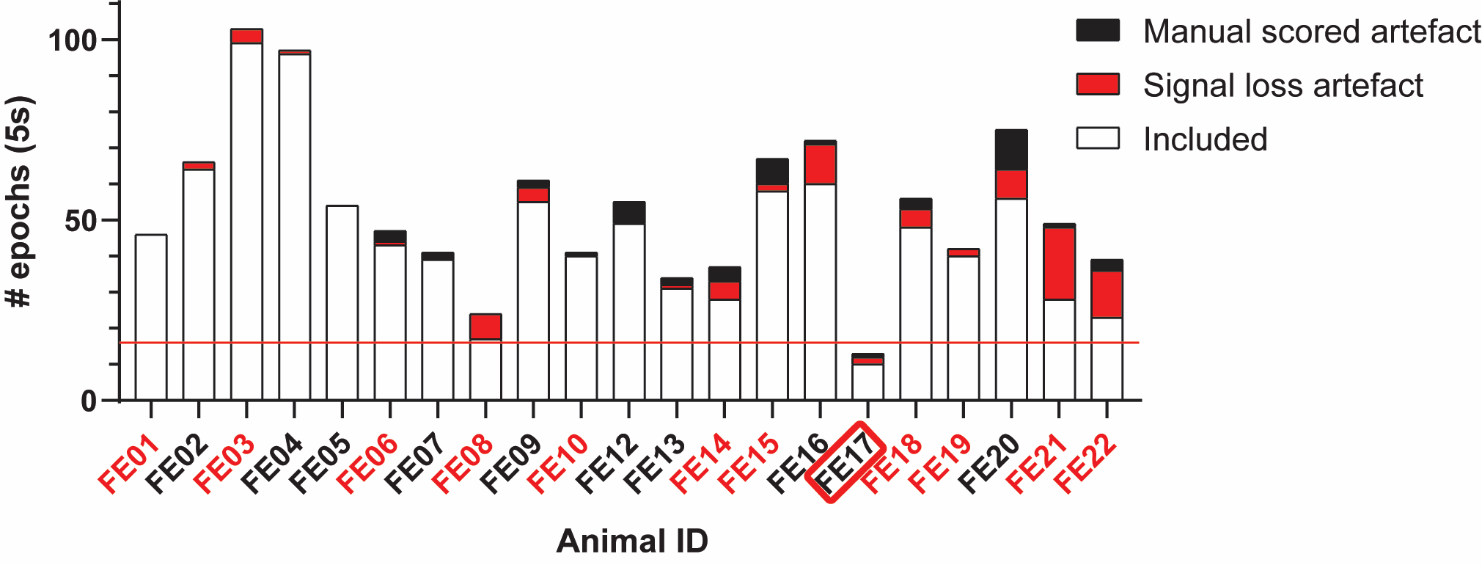
**

**Supplementary figure 1. Overview of the number of resting-state epochs per animal in Experiment 1.** The total number of 5-second resting-state epochs is plotted per animal. Both signal loss and manual artefact rejection are indicated. The horizontal red line is the minimum required amount of data (16 epochs, 80 seconds). One animal (FE17) did not reach this minimum and was excluded from the analyses. FE11 is missing as this recording could not be scored for resting-state due to low quality EMG electrodes. WT animal IDs are depicted in black; KO animal IDs are depicted in red.


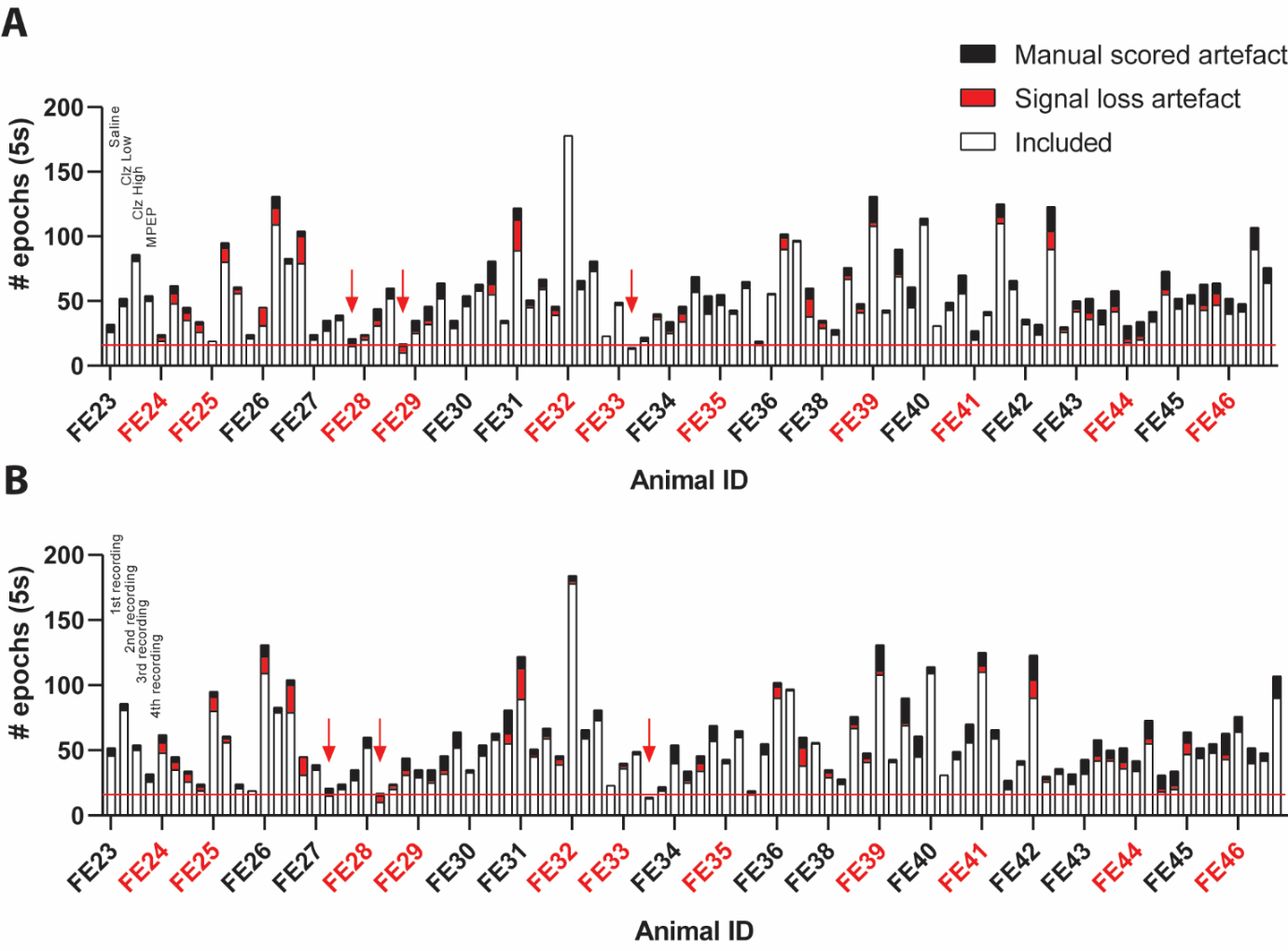


**Supplementary figure 2. Overview of the number of resting-state epochs per animal in Experiment 2.** The total number of 5-second resting-state epochs is plotted per animal. The recordings are either ordered by pharmacological compound (A) or chronologically (B). Both signal loss and manual artefact rejection are indicated. The horizontal red line is the minimum required amount of data (16 epochs, 80 seconds). Three recordings (indicated by red arrows) were excluded from the analysis because they did not contain sufficient resting-state data. FE37 is missing since this animal died from a post-operative cardiac arrest. WT animal IDs are depicted in black; KO animal IDs are depicted in red.


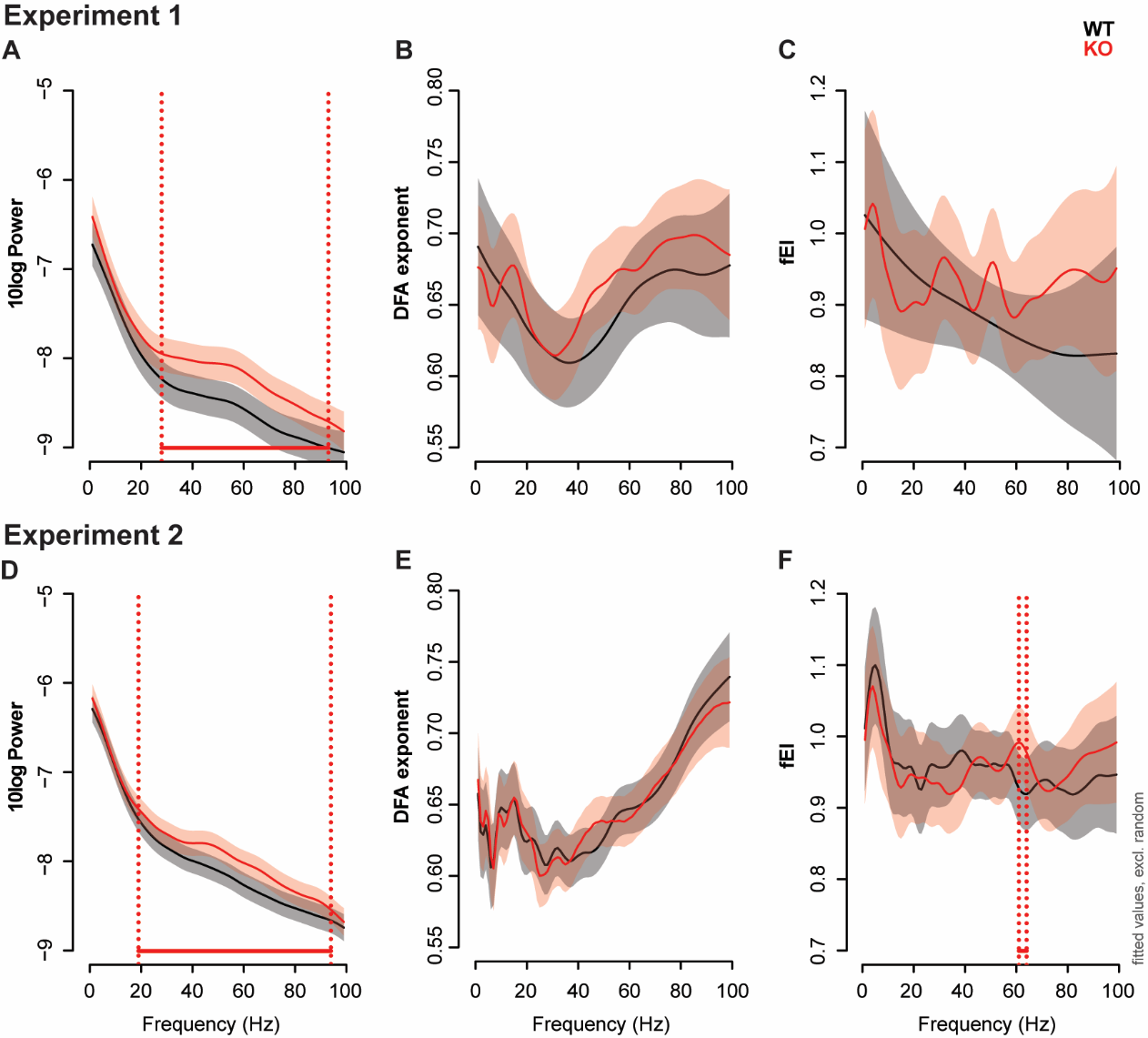


**Supplementary figure 3. Fmr1 KO2 mice show no evidence for E/I alterations in frontal cortex.** Resting-state EEG was extracted from baseline recordings to assess network functionality in the Fmr1 KO2 model. EEG power (A,B), long-range temporal correlations (LRTC) (C,D), and the fEI ratio (E,F) were computed on the resting-state segments in 1-Hz frequency bins in the 1–99 Hz range. General additive mixed models (GAMMs) were used to model the effect of genotype and pharmacological exposure (Experiment 2). The figures show the fitted values excluding random effect factors of the frontal recording electrodes. Since there was no significant interaction between genotype and pharmacological exposure, the fitted values are shown for the saline condition for Experiment 2. Frequency ranges where the confidence bands of the difference wave between WT and KO (not shown) do not cross zero, were considered significant and are marked red in the figures. Experiment 1: N_WT_ = 9, N_KO_ = 11; Experiment 2: N_WT_ = 12, N_KO_ = 11.


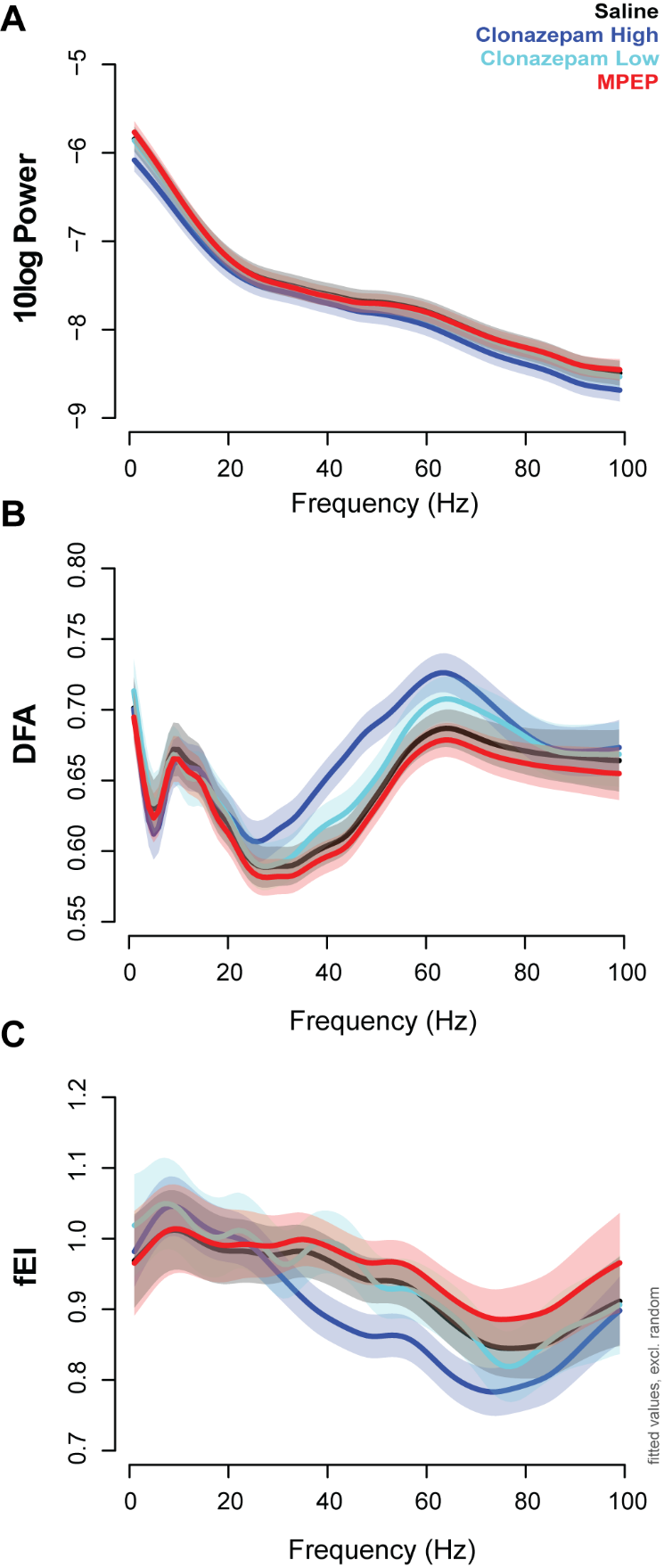


**Supplementary figure 4. The effect of exposure to pharmacological compounds on network function in FMR1 KO mice.** Similar effects of pharmacological exposure were seen in KO mice as compared to WT mice (plotted in figure 3). To create these figures, resting-state EEG was extracted from baseline recordings to assess network functionality in the Fmr1 KO2 model after administration of low (0.1 mg/kg) or high (0.25 mg/kg) dose clonazepam and MPEP (25 mg/kg). EEG power (A), long-range temporal correlations (LRTC) (B), and the fEI ratio (C) were computed in 1-Hz frequency bins in the 1–99 Hz range. General additive mixed models (GAMMs) were used to model the effect of genotype and pharmacological exposure. The figures show the fitted values, without random effects of the temporal cortex recording electrodes. N = 11 (NClzL & NMPEP = 10).

**
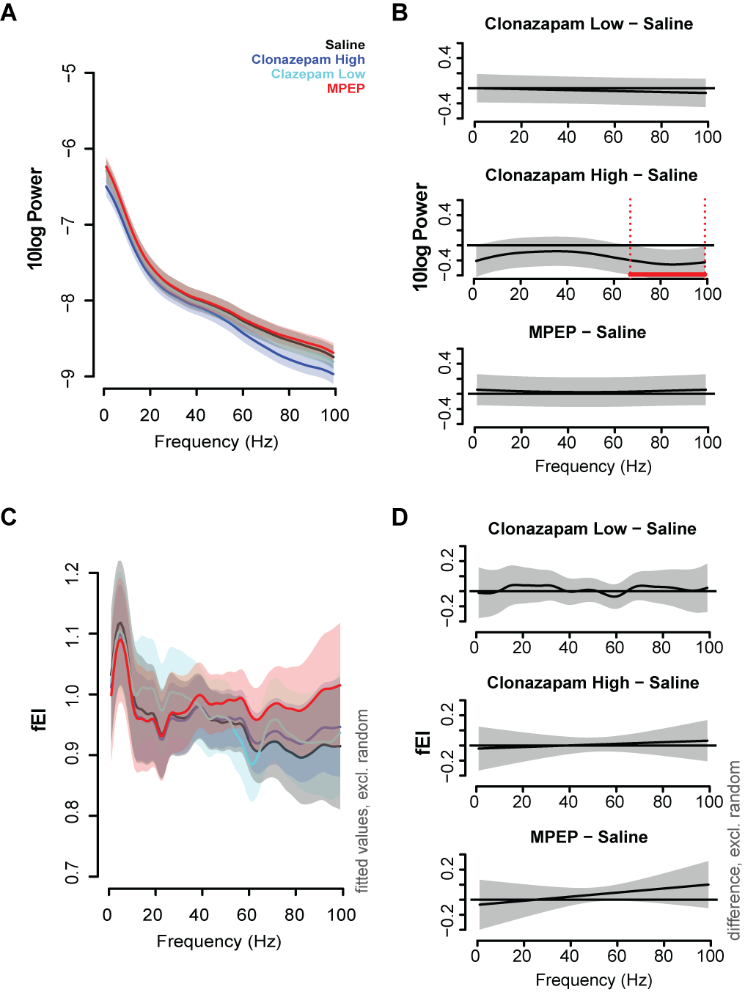
**

**Supplementary figure 5. Pharmacological compounds have a small effect on the network function outcomes in the frontal cortex.** Resting-state EEG was extracted from baseline recordings to assess network functionality in the Fmr1 KO2 model after administration of low (0.1 mg/kg) or high (0.25 mg/kg) dose clonazepam and MPEP (25 mg/kg). EEG power (A,B) and fEI (C,D) were computed on the resting-state segments in 1-Hz frequency bins in the 1–99 Hz range. General additive mixed models (GAMMs) were used to model the effect of genotype and pharmacological exposure. The figures show the fitted values, without random effects of the frontal recording electrodes. Since there was no interaction between genotype and pharmacological exposure, the figure shows the fitted values for WT mice. GAMMs showed also no main effect of the pharmacological exposure for the DFA, this data is therefore not shown. Frequency ranges where the confidence bands of the difference wave between WT and KO (not shown) do not cross zero, were considered significant and marked red in the figures. N_WT_ = 12 (N_WT-MPEP_ = 11); N_KO_ = 11 (N_KO-ClzL_ & N_KO-MPEP_ = 10).
